# An adjustable fetal weight standard for twins: a statistical modeling study

**DOI:** 10.1186/s12916-015-0401-9

**Published:** 2015-07-03

**Authors:** Jun Zhang, Rafael Mikolajczyk, Xiaoping Lei, Luming Sun, Hongping Yu, Weiwei Cheng

**Affiliations:** MOE-Shanghai Key Laboratory of Children’s Environmental Health, Xinhua Hospital, Shanghai Jiao Tong University School of Medicine, 1665 Kong Jiang Road, Shanghai, 200092 China; EMSE – Epidemiological and statistical Methods Research Group, Helmholtz Centre for Infection Research, Braunschweig, Germany; Fetal Medicine Unit & Prenatal Diagnosis Center, Department of Obstetrics, Shanghai First Maternity and Infant Hospital, Tongji University School of Medicine, Shanghai, China; School of Public Health, Guilin Medical College, Guangxi, China; Obstetrics Department, International Peace Maternity & Child Health Hospital, Shanghai Jiaotong University, Shanghai, China

**Keywords:** Adjustable, Fetal, Standard, Twins, Weight

## Abstract

**Background:**

It is a common practice to use a singleton fetal growth standard to assess twin growth. We aim to create a twin fetal weight standard which is also adjustable for race/ethnicity and other factors.

**Methods:**

Over half a million twin births of low risk pregnancies in the US, from 1995 to 2004, were used to construct a fetal weight standard. We used the Hadlock’s fetal growth standard and the proportionality principle to make the standard adjustable for other factors such as race/ethnicity. We validated the standard in different race/ethnicities in the US and against previously published curves from around the world.

**Results:**

The adjustable fetal weight standard has an excellent match with the observed birthweight data in non-Hispanic White, non-Hispanic Black, Hispanics, and Asian from 24 to 38 weeks gestation. It also had a very good fit with cross-sectional data from Australia and Norway, and a longitudinal standard from Brazil. However, our model-based 10th and 90th percentiles differed substantially from studies in Japan and US that used the last menstrual period for estimate of gestational age.

**Conclusion:**

The adjustable fetal weight standard for twins is a flexible tool and can be used in different populations.

**Electronic supplementary material:**

The online version of this article (doi:10.1186/s12916-015-0401-9) contains supplementary material, which is available to authorized users.

## Background

The use of a fetal growth standard for singleton pregnancies in order to assess twin growth is common practice. While twin and singleton fetuses may follow a similar growth pattern during the first and second trimesters [1], studies have shown that their growth patterns diverge in the third trimester [2–4]. A study by Joseph et al. [5] convincingly demonstrated that singletons and twins need separate standards in order to evaluate their growth appropriately. Furthermore, similar to singletons, there are racial/ethnic differences in fetal sizes among twins [6, 7]. One standard may not fit all populations without misclassification of small- and large-for-gestational-age fetuses. Therefore, an ideal twin standard should also be able to take such factors as race into account. However, twin pregnancies are less than 2% of all pregnancies, which makes it difficult to establish a twin fetal growth standard, let alone to have customized standards suitable for various populations and institutions. The purpose of this study is to create a twin fetal weight standard that is also adjustable for race/ethnicity and other factors.

## Methods

### Creation of an adjustable fetal weight standard for twins

The method to create an adjustable fetal weight standard for twins is the same as our previous work for singletons [8]. However, in contrast to the singleton adjustable standard, which anchors the Hadlock’s curve to a mean birth weight at 40 completed weeks, the twin standard anchors to a mean birthweight at 37 completed weeks of a particular population. Specifically, we first adapted the widely accepted, ultrasound-based fetal growth standard proposed by Hadlock et al. [9]. Its formula to calculate median fetal weight for each gestational week [fetal weight (g) = exp(0.578 + 0.332×GA – 0.00354×GA^2^)] was used as the base for fetal growth pattern. GA refers to gestational age in exact weeks (e.g., 36 weeks + 5 days = 36.7 weeks). Hadlock et al. [9] used ultrasound measurements between 10 and 41 weeks gestation of 392 singleton pregnant women of the European Continental Ancestry Group living in the USA, i.e., White, to create this optimum growth equation. They also showed that the statistical variation of fetal weight in a given gestational week was a constant fraction of the mean. On the basis of this information, they provided fetal-weight percentiles for each gestational week. They also showed that the statistical variation of fetal weight in a given gestational week was a constant fraction of the mean. On the basis of this information, they provided fetal-weight percentiles for each gestational week.

Second, we adopted the proportionality principle proposed by Gardosi et al. [10], which assumes that individual weight can be expressed as a percentage of the expected weight based on Hadlock’s growth equation. We assumed that Hadlock’s growth equation could be used to derive percentiles of fetal weight in a given gestational week for a different population by anchorage of the formula to a mean birthweight at 37 complete weeks. We first obtained the mean birthweight at 37.5 weeks of low-risk twins in a White population in the US (described below). This mean birthweight (MW.GA = 37) was then divided by the constant of 3,133 g, which is the mean birthweight at 37.5 weeks of gestation in Hadlock’s fetal growth equation. The obtained ratio was assumed to be constant across gestation, i.e., if the mean birthweight at 37 weeks in a particular population was 0.86 (or 86 %) of those of White fetuses in the US, then it was also 0.86 at 34 weeks. Next, we multiplied fetal-weight estimates based on Hadlock’s reference for each gestational week by this ratio and obtained mean fetal weight estimates across gestation for the specific population to make the Hadlock function adjustable according to the size of twin births at term.

Following Hadlock’s method, we assume that the standard deviation (SD) expressed in percent of the mean weight is constant across gestation. Based on normal distribution, corresponding percentiles can be calculated. A complete fetal weight standard for twins, therefore, is created. It is adjustable for any population or institution, which can use its own mean birth weight at 37.5 weeks (i.e., the mean of all births at 37 completed week) as an anchoring point for the fetal weight standard. For easy use, we have created an Excel®-based software that can be readily applied (Additional file 1).

### Application to the US population

To create and validate our adjustable standard, we first used the US Linked Live Birth and Infant Death files from 1995 to 2004 [11]. These data present the national live birth registry linked to infant deaths and compiled by the National Center for Health Statistics, US Centers for Disease Control and Prevention; detailed description of this dataset is provided elsewhere [12]. Available information in this dataset included demographic characteristics of mothers, obstetric history, birth outcomes, and infant death.

For most women (86.8 %), two different types of gestational age estimates were recorded on the birth certificate in these data: clinical estimate (CE) and gestational age based on self-reported last menstrual period (LMP). Deficiencies of LMP-based gestational age are well established [13]. Recently, Qin et al. [14] used a simple method in which the CE of gestational age is substituted for LMP-based gestational age when the difference between the two estimates is greater than 2 weeks (LMP/CE method). They demonstrated that the LMP/CE method, when compared to the other techniques, almost eliminated the second mode in gestational age distribution. Thus, this method appears to be effective in correcting large errors in gestational age estimates. It has the further benefit that records are reclassified, rather than excluded altogether. Given these strengths, we adopted LMP/CE method for purposes of our analysis; 10.7 % of women had a replacement.

In the current analysis, we first separated the study population by race/ethnicity (non-Hispanic White, non-Hispanic Black, Hispanics, and Asian). Second, we calculated mean birthweight and SD at 37 completed weeks for each race/ethnicity (Table 1). Using the adjustable standard weight percentiles calculator, we produced race-specific fetal weight percentiles.

**Table 1:** Mean birthweight at 37 weeks gestation among different race/ethnicity twins in the US 1995–2004

Race/ethnicity	N	Percentage	Mean birthweight (g)	Standard deviation	SD/mean (%)
White	380,295	70.9	2,727	374	13.7
Black	90,228	16.8	2,605	378	14.5
Hispanic	41,905	7.8	2,663	381	14.3
Asian	13,189	2.5	2,581	372	14.4
Other	10,892	2.0			
Total	536,479	100.0			

**Fig. 1:**
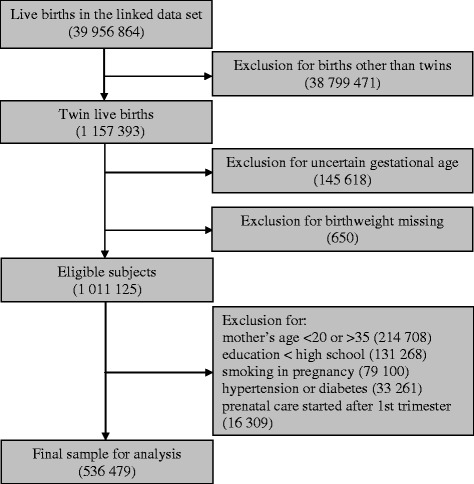
Subject selection process, the US Linked Live Birth and Infant Death Database, 1995–2004

There were 1,157,393 twins in the linked 1995–2004 birth and infant death dataset (Fig. 1). We restricted our analysis to low risk twin live births, i.e., women with reliable gestational age and birthweight, maternal age between 20 and 35 years, high school graduate or higher education, non-smokers, no hypertensive disorders in pregnancy or pre-existing or gestational diabetes, and prenatal care started in first trimester, leaving 536,479 twins for the final analysis.

### Comparison with other standards

We compared our standard with three birthweight-based twin standards [15–17], two cross-sectional ultrasound-based fetal weight standards [3, 18], and two longitudinal fetal weight standards [19, 20]. We first chose the mean birthweight and SD at 37 weeks from each study and calculated an adjusted fetal weight standard using the above program. Then, we plotted our growth curve against the curves in these studies and compared the differences.

### Ethics statement

Data for this analysis were obtained from anonymous data rendering an exemption of ethical approval by the Shanghai Xinhua Hospital Research Ethics Board.

## Results

**Fig. 2:**
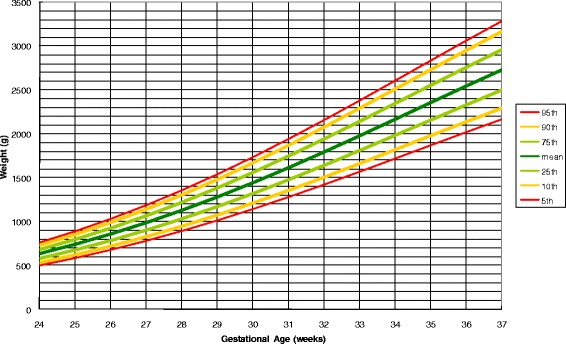
An adjustable fetal weight standard for twins

Figure 2 illustrates the adjustable fetal weight standard for twins. We applied this standard to different populations and compared with standards published previously.

**Fig. 3:**
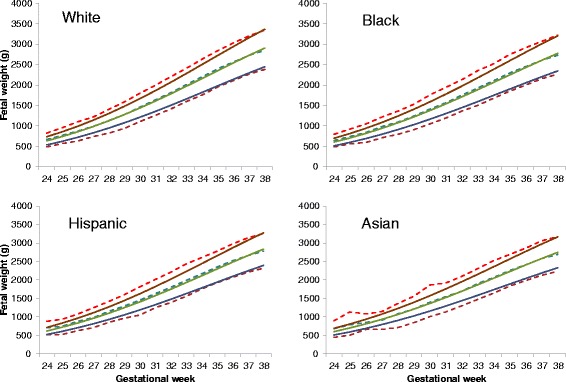
Observed 10th, 50th, and 90th percentiles of birthweight for gestational week (dashed lines) by race/ethnicity in a US twin population, 1995–2004, in comparison to the corresponding adjusted fetal weight standard (solid lines)

Table 1 presents the mean birthweight at 37 weeks gestation by different race/ethnic groups in the US – Black and Asian twins had mean birthweights approximately 100 and 150 g lower than White twins at 37 weeks gestation. We then compared the observed birthweight by gestational week with the adjusted standard in White, Black, Hispanic, and Asian twin births. Figure 3 shows that the adjustable standard at the 50th percentile matched the mean observed birthweight curves very well for all races/ethnicities in the US from 24 to 38 weeks gestation. However, the adjustable standard tended to have a narrower range at the 10th and 90th percentiles than the observed data.

**Fig. 4:**
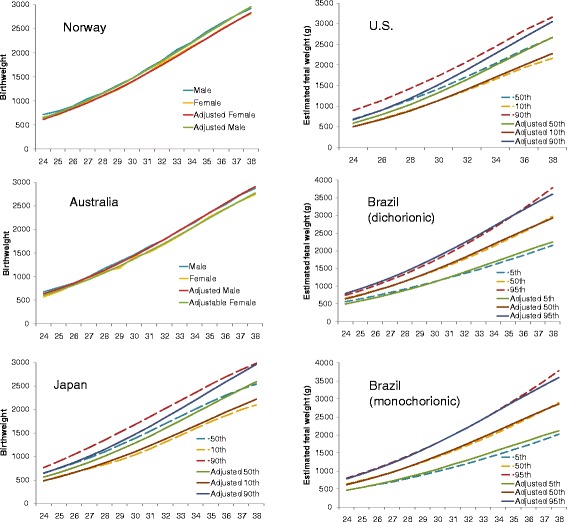
Comparisons between observed birthweight (Norway [15], Australia [16], Japan [17]), estimated fetal weight (US [3], Brazil dichorionic and monochorionic [19]), and adjusted fetal weight by the adjustable fetal weight standard for twins in five previous studies. The 50th percentile birthweight was selected in the Norwegian and Australian studies [15, 16]

We also compared twin birthweight references from Australia, Norway, and Japan. Figure 4 (left column) shows that the observed and adjusted curves overlapped for both males and females in Australia and Norway. However, the curves differed quite substantially for Japan. Although the 10th percentile curves were similar, the 50th and 90th percentiles curves were much higher than those of the adjustable standard, particularly in early gestation. Furthermore, in the Japanese data the percentile ranges were much wider in early gestation, lacking a typical gramophone shape. This phenomenon was also observed in a US cross-sectional study using clinically recorded estimated fetal weight (Fig. 4, right column). In contrast, the adjustable standard matched well with a Brazilian cross-sectional study with estimated fetal weight in both monochorionic and dichorionic twins. It is interesting to note that the 5th and 95th limits were appreciably wider for the monochorionic than the dichorionic twins.

**Fig. 5:**
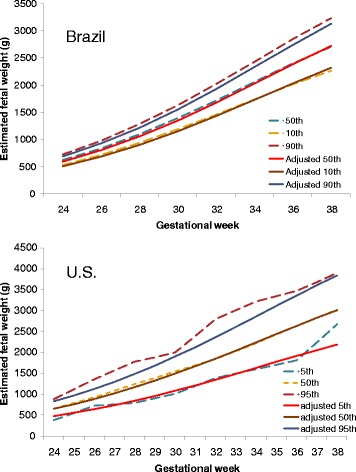
Comparisons between observed and adjusted fetal growth curve in two longitudinal studies [19, 20]

Finally, we compared our standard with two longitudinal fetal growth standards from Brazil and the US (Fig. 5) [19, 20]. The Brazilian study serially measured 125 low-risk twin sets every 3 weeks, on average, from 14 to 38 weeks gestation. Multilevel regression analysis was performed on normalized data. Our adjustable curves matched well with the longitudinal standard, except that the Brazilian standard had a higher 90th percentile curve than the adjustable standard. Yarkoni et al. [20] conducted a small longitudinal study in 35 healthy women with normal twin pregnancies in the US. Ultrasound measures were taken every 3 weeks from 15 weeks gestation to delivery. Noticeably, the 5th and 95th limits were less stable probably due to the small sample size. However, the 50th percentile curve was almost identical to ours.

## Discussion

Based on the Hadlock fetal growth standard for singletons and the proportionality principle, we created an adjustable fetal weight standard for twins. It can be easily adjusted to the local population and individual institutions. We found that the adjusted standard matched with Australian, Brazilian, Norwegian, and American twin birth standards well, which validates our method.

It has been shown that normal fetal growth follows an intrinsic pattern that can be expressed by a mathematical function, i.e., the proportionality function [10]. Numerous studies have also demonstrated that the distribution of estimated fetal weight at each gestational week is close to normal [2, 9, 10], which lends the opportunity to simply use the SD of the normal distribution to calculate the full spectrum of percentiles. These two principles provided the foundation of our work. Our findings were validated through comparisons with observed and published data, particularly with the longitudinal twin standards [19, 20].

While the adjusted curves matched the observed and published data well in general, several issues are worth discussing. First, studies have shown that singleton and twin growth patterns diverge in late pregnancy [2–4]. Why then did the adjustable twin standard that is derived from the singleton growth equation match the observed data seamlessly? One explanation is that twins may have a constantly slower growth velocity than singletons. At early gestation, when fetuses are small, singleton and twin curves are very close. As the fetuses grow, the difference in fetal size emerges and becomes larger with gestation.

Second, studies of singleton gestation have shown that preterm, especially very preterm births (<34 weeks of gestation), are more likely to be growth restricted. Their mean birthweight, therefore, is substantially below the mean estimated fetal weight at a given gestation [21]. However, in twins, this discrepancy does not seem to be substantial. We found that the adjusted mean fetal weight curve matched the birthweight curves in the Australian, Norwegian, and American data, even in very preterm births. This was further confirmed by the comparison with the longitudinal ultrasound twin standards [19, 20], which are considered the “gold standard” herein. These findings suggest that preterm twins may not necessarily be severely growth restricted.

Nonetheless, the 10th and 90th percentile curves did differ substantially between observed data in Japan (birthweight) [17] and US (estimated fetal weight) [3] and our adjusted standard for these countries – the discrepancy was particularly large in early gestation. By closely examining the observed data, we found that the typical gramophone shape of fetal growth curves was not clear in these data, suggesting that errors in gestational age may have had a substantial impact on the created percentiles of birthweight. Nevertheless, one must bear in mind that these studies used retrospective clinical data and mostly relied on the LMP for dating of the pregnancy. Previous research has extensively documented that errors in gestational age can cause the above phenomenon [22]. Indeed, our percentile patterns were much closer to those in longitudinal studies with careful dating and measuring [19, 20] and an obvious gramophone shape. It is worth noting that specifically designed prospective studies with carefully measured ultrasound data in twins are invaluable in validating our tool. The curves presented herein fit well with these.

In addition, we would like to point out that our method to create a fetal growth standard can be used for both standard and population reference. When the mean birthweight and SD at 37 weeks are obtained from population-based data, the adjusted curves are a population reference. On the other hand, if the mean birthweight and SD at 37 weeks are obtained from low-risk pregnancies with normally grown fetuses, the curves are a standard [23].

Finally, a recent fetal growth study collected data in healthy, well-nourished women living in environments with minimal constraints on fetal growth, across eight geographically diverse urban areas worldwide [24]. The results showed substantial variations in fetal size at birth among races/countries. For example, the singleton mean birthweight at term in India was 2.9 kg, while the corresponding birthweight in the UK was 3.5 kg [24], suggesting that there are substantial differences in fetal growth potential among races that cannot be explained by environment. Such evidence argues for a race-specific twin fetal growth standard. Nevertheless, the ultrasound estimation of fetal weight has an intrinsic error. In research studies, an error <10% on average can be achieved [25], but in daily clinical practice this error could be larger. Thus, one may argue that adjusting for racial variation in twin fetal size may not be necessary. Indeed, the benefit of a race-specific standard for twin fetal growth may be small in countries and institutions with a predominately homogeneous Caucasian population. The benefit is probably more prominent for international comparisons [8].

## Conclusion

It is common practice to use a singleton fetal growth standard to assess twin growth, which could substantially misclassify a high proportion of twins as growth restricted. Furthermore, there are differences in twin fetal size among races and populations. The adjustable fetal growth standard for twins is a flexible tool and can be used in different populations as a standard or population reference. It can reduce the misclassification of abnormal fetal growth and provide more accurate fetal assessment, particularly in international comparisons. For easy use, we have created an Excel-based software that can be readily applied (Additional file 1). Further validation with carefully conducted (either cross-sectional or longitudinal in study design), prospectively collected ultrasound-based fetal growth standards for twins in various populations is warranted.

## Additional file

Additional file 1:
**Adjustable fetal weight standard for twins.**

## Abbreviations

CE: Clinical estimate
LMP: Last menstrual period
SD: Standard deviation
